# Walking Recognition in Mobile Devices

**DOI:** 10.3390/s20041189

**Published:** 2020-02-21

**Authors:** Fernando E. Casado, Germán Rodríguez, Roberto Iglesias, Carlos V. Regueiro, Senén Barro, Adrián Canedo-Rodríguez

**Affiliations:** 1 CiTIUS (Centro Singular de Investigación en Tecnoloxías Intelixentes), Universidade de Santiago de Compostela, 15782 Santiago de Compostela, Spain; senen.barro@usc.es; 2 Situm Technologies S.L., 15782 Santiago de Compostela, Spain; german.rodriguez@situm.es (G.R.); adrian.canedo@situm.es (A.C.-R.); 3 CITIC, Computer Architecture Group, Universidade da Coruña, 15071 A Coruña, Spain; carlos.vazquez.regueiro@udc.es

**Keywords:** walking recognition, activity recognition, smartphones, inertial sensor fusion, pattern classification, time series classification.

## Abstract

Presently, smartphones are used more and more for purposes that have nothing to do with phone calls or simple data transfers. One example is the recognition of human activity, which is relevant information for many applications in the domains of medical diagnosis, elderly assistance, indoor localization, and navigation. The information captured by the inertial sensors of the phone (accelerometer, gyroscope, and magnetometer) can be analyzed to determine the activity performed by the person who is carrying the device, in particular in the activity of walking. Nevertheless, the development of a standalone application able to detect the walking activity starting only from the data provided by these inertial sensors is a complex task. This complexity lies in the hardware disparity, noise on data, and mostly the many movements that the smartphone can experience and which have nothing to do with the physical displacement of the owner. In this work, we explore and compare several approaches for identifying the walking activity. We categorize them into two main groups: the first one uses features extracted from the inertial data, whereas the second one analyzes the characteristic shape of the time series made up of the sensors readings. Due to the lack of public datasets of inertial data from smartphones for the recognition of human activity under no constraints, we collected data from 77 different people who were not connected to this research. Using this dataset, which we published online, we performed an extensive experimental validation and comparison of our proposals.

## 1. Introduction

Our society is more and more surrounded by devices—smartphones, tablets, wearables, “things” from the Internet of Things (IoT), etc.—which are rapidly transforming us, changing the way we live and interact with each other. The gradual incorporation of new sensors on these devices provides significant opportunities towards the development of applications that use these sensors in an ever-increasing number of domains: healthcare, sport, education, leisure, social interaction, etc. Thus, inertial sensors are being used in the smartphones to monitor human activity and, in particular, the action of walking. The information about whether the user is walking or not is very valuable for many applications, such as medicine (detection of certain pathologies) [1], biometric identification (recognition of the owner of the device based on his or her characteristic gait) [2,3,4,5], elderly assistance [6], emergency services [7], monitoring systems [8] and pedestrian indoor localization [9,10,11].

In the particular case of pedestrian indoor localization, recognizing the activity of walking using inertial sensors is essential, since other alternatives such as the Global Navigation Satellite System (GNSS) do not work indoors. Although other sensor modalities, such as infrared, ultrasound, magnetic field, WiFi or BlueTooth [12,13,14], have been used to detect the displacement of a person indoors, the combination of the information provided by these sensors together with the recognition of walking using the accelerometer, magnetometer and gyroscope (IMU) has been proved to be the best option to significantly increase the performance of indoor localization.

Other sensors and processing strategies were applied to identify the activity of walking, such as cameras for visual odometry or pressure sensors attached to the shoes [9]. These approaches involve additional hardware attached to the body (foot, arm, trunk, etc. [10,11,15]), so that their processing is simpler and the outcome more reliable. However, the placement of sensors on the body or clothing greatly restricts their applicability. Instead, using the inertial sensors that the vast majority of smartphones already have (accelerometer, gyroscope and magnetometer) is much more attractive, since they present fewer restrictions and most people already carry this kind of devices.

**Figure 1:**
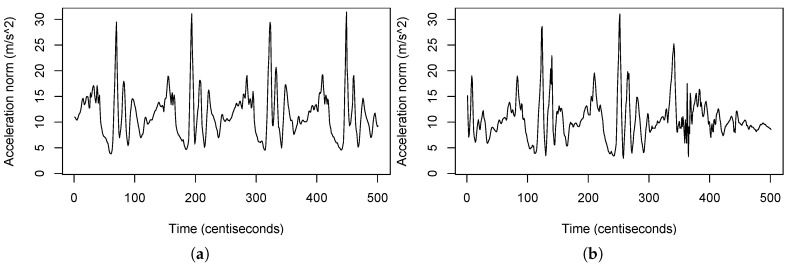
Norm of the acceleration experienced by a mobile phone when its owner is walking (**a**), and not walking, but gesticulating with the mobile in his/her hand (**b**).

One of the biggest advantages of using the inertial sensors of the smartphone for walking recognition is that very little physical infrastructure is required for these kind systems to function. Nevertheless, achieving a robust recognition system for this task is more complex than it might seem. It is relatively easy to detect the walking activity and even count the number of steps given when people walks ideally carrying the device in the palm of his/her hand, facing upwards and without moving it. However, the situation becomes more complicated in real life, since the orientation of the smartphone with respect to the user, as well as its placement (hand, pocket, ear, bag, etc.), can constantly change as the person moves [16]. Getting a robust classification regardless of the device carrying mode and its orientation is challenging. This kind of devices experience a large variety of motions producing different patterns in the signal. Frequently, we obtain similar patterns for movements or actions that have nothing to do with walking, which makes the recognition of this activity a complex task. Figure 1 shows the complexity of this problem with a real example. In this figure we can see the norm of the acceleration experienced by a mobile phone while its owner is doing two different activities. The person and the device are the same in both cases. In Figure 1a, we can see the signal obtained when the person is walking with the mobile in the pocket. The impacts of the feet touching the floor are reflected in the signal as local maximum points. Please note that identifying and counting these peaks, for example applying a simple peak-valley algorithm [17], would easily allow counting the number of steps. Figure 1b shows the acceleration experienced by the mobile when the user is standing still with the mobile in his hand, without walking, but gesticulating with the arms in a natural way. Unfortunately, in this case, the peaks of the signal do not correspond to steps, so the aforementioned peak-valley algorithm would detect false positives.

In this work, we carried out an exhaustive analysis and development of different methodologies to solve the problem of walking recognition in mobile devices. We made an extensive review of the state of the art and we explored and compared several approaches based on different machine learning techniques that allow a robust detection of the walking activity in mobile phones. We categorize these approaches into two main groups: (1) feature-based classification and (2) shape-based classification.

The paper is structured as follows: Section 2 gives a review of the state of the art. Section 3 details some tasks of preprocessing the inertial data that are necessary. Section 4 describes our proposals for walking recognition. Section 5 introduces the database that we used for ground truth and shows the experimental analysis of our proposals. Finally, some conclusions are presented in Section 6.

## 2. State of the Art

Thanks to technological progress in recent years, it is possible to develop inertial sensors of very small size, ideal for integration into smartphones. Taking advantage of their potential, several solutions have been developed in walking recognition. In most of the works, the position of the device is kept fixed (foot, arm, trunk, etc.) [10,11,15], because any change in position could result in a drop in performance. This is not desirable because in real world people carry their smartphones in different ways (hand, pocket, handbag, etc.) and they do not keep them in a static position. Recently, more research has been carried out on activity recognition without restricting the position of the phone. Some authors use the magnitude of the acceleration as input to make the signal invariant to orientation [18,19]. This may work well in controlled situations when the user walks normally, but the resulting signal is contaminated with excitations unrelated to walking when using the smartphone in different ways.

### 2.1. Heuristic Methods

A simple approach to identify the activity of walking is counting the steps given by the user of the smartphone. The most common way to perform that count is the a heuristic solution present in many pedometer applications. It consists of using a peak-valley detector [17] to identify events, such as heel strikes, where the impacts of the feet are reflected in the vertical component of the acceleration. In this way, a step corresponds to a signal segment in which there is a peak (local maximum exceeding a threshold) followed by a valley (local minimum below a threshold). However, this type of algorithm is susceptible to detect any motion produced within the expected range of frequencies (e.g., situations like the one in Figure 1b), which makes it prone to commit false positives. Moreover, it also often has problems to detect changes in the walking speed [20]. Due to this, it is necessary another complementary module to perform a real-time filtering of those parts of the signal that capture some kind of movement in the device, but have nothing to do with walking [21]. This is a challenge because of the high perceptual aliasing (i.e., the existence of signals that are very similar to each other but caused by completely different movements).

### 2.2. Feature-Based Approach

One robust way to identify the walking activity is by extracting relevant features from the inertial data and using them to train a classifier. Bradjic et al. [20] conducted an experiment in which they evaluated different classifiers using several features in time and frequency domains extracted from the inertial data. They employed a large dataset of 130 sensor traces of 27 different users walking and performing different activities while carrying a smartphone. Even though the accuracies they reported are high, these algorithms still present a high number of false positives when the phone is being moved but the user is not walking. Susi et al. [19] tried not only to identify walking activity, but also classify the motion mode of the device (static, handheld texting, swinging, etc.) by extracting time and frequency domain features and training a decision tree. They reported a good performance, but they only evaluated their proposal in controlled tests walking while texting and swinging. Zou et al. [22] used deep learning techniques to learn and model the gait biometrics. In particular, features from time and frequency domains were successfully abstracted by combining a convolutional neural network with a recurrent neural network. In their experiments, two datasets collected by 118 different people were used for evaluations achieving high accuracies. However, their approach is not valid for real time operation in mobile devices.

### 2.3. Shape-Based Approach

Another way of dealing with the classification problem is by comparing the shape of the inertial signals. For that, it is assumed that it is possible to distinguish the activity of walking from that of not walking by simply observing the characteristic signal of the accelerometer. This is mainly because, as it was pointed out before, when walking, the acceleration signal shows a recurring pattern, also known as step cycle. The event that is often used to mark the beginning of the cycle is the heel strike of the swing leg [3,4], which is the impact produced when both feet are on the ground and they are farthest from each other. When this impact occurs, a local minimum should be observed in the vertical component of the acceleration. Thus, the step cycle can be detected by extracting the timestamps of the heel strikes. However, identifying the step cycle is challenging because the accelerometer readings may be distorted due to the irregular movement of the user’s body or changes in walking speed. Then, it is necessary to find a match between the step cycle candidates and one or more patterns selected in advance using a distance metric. Euclidean distance is a simple metric, but it was observed that it is very sensitive to noise and distortion and very similar patterns can be separated by very large distances if the data items are not aligned [23]. A better option is to use warping distances, such as dynamic time warping (DTW) [24], longest common subsequence (LCSS) [25] or edit distance on real sequence (EDR) [23]. The main drawback of this approach is that if the step candidates are misidentified during the signal division process, the subsequent matching with the reference patterns is compromised. Moreover, there is a need to arrange a set of reference patterns, which must be chosen in advance and, most likely, manually.

Most of the experimentation in the bibliography only uses data of people walking, so it is not clear how these algorithms would behave in terms of false positives when the person uses the device without walking. In addition, there is a tendency to evaluate the performance of these algorithms to measure the total traveled distance or the total number of detected steps, but there is no evaluation of whether each single step being detected is true or not. We believe that such a thorough evaluation is important because false positives and false negatives can cancel each other and mask the real performance of the system in short time intervals.

## 3. Signal Preprocessing

The walking recognition is performed using the signals provided by the tri-axis accelerometer and the tri-axis gyroscope in a mobile phone, and which respectively measure the acceleration and the angular velocity of the device with respect to the device frame, which is the reference system linked to the phone as it is defined relative to its screen. The output of these sensors is a 6-dimensional time series composed by the accelerometer output $a_{t}\in R^{3}$ and the gyroscope output $\omega_{t}\in R^{3}$:

(1) $$\left[\begin{array}{c} a_{t}\\ \omega_{t} \end{array}\right],$$

where *t* represents the temporal index of the signal. The sampling frequency in our case is 100 Hz.

Due to their low quality, the sensors embedded in most common mobile devices are strongly affected by errors degrading their accuracy [26]. To deal with such errors, we carry out a specific signal preprocessing stage, making transformations on the raw sensor data; First, we estimate its orientation with respect to an inertial reference system, the Earth frame, the axes of which always points towards the same points (with respect to the Earth). Second, we can estimate the linear acceleration which is being experienced by the phone in the Earth reference system and, finally, we can filter and center the resultant signal for noise reduction.

### 3.1. Attitude Estimation

As the mobile phone can be carried in any position and orientation, we need to know its attitude, or 3D orientation, to extract the vertical component of the acceleration, which contains the information of the heel strikes. The magnitude of the acceleration can be used in some scenarios, since it is independent of phone orientation, but it is not robust enough since it can be affected by accelerations not related to walking.

To understand this stage we must be aware of the existence of two reference systems: (1) the local one (device frame), linked to the phone, and (2) the inertial reference system (Earth frame). In the case of the inertial-Earth frame, we work with a frame analogous to the East North Up (ENU) coordinate system [27], in which the *x*-axis points toward the East, the *y*-axis points towards the North Magnetic Pole and the *z*-axis points in the opposite direction of the gravitational force. The readings of the sensors are provided in the body frame, and therefore it is convenient to project them into the Earth frame in order to estimate the movement of the person who carries the mobile. Hence, it is necessary to know the orientation (attitude) of the mobile with respect to the inertial Earth frame.

To represent this orientation we use quaternions, because of their many advantages over other representations [28]. A quaternion is a four-dimensional vector that represents the transformation among two reference systems, *A* and *B*, as a rotation of an angle θ around a three-dimensional axis $u=[u_{x}\;u_{y}\;u_{z}]$, such that:

(2) $${}_{B}^{A}q=\left[\begin{array}{c} q_{0}\\ q_{1}\\ q_{2}\\ q_{3} \end{array}\right]=\left[\begin{array}{c} cos\theta \\ sin\theta \;u_{x}\\ sin\theta \;u_{y}\\ sin\theta \;u_{z} \end{array}\right],$$

being ${}_{B}^{A}q$ the normalized quaternion that represents the orientation of frame *B* relative to frame *A*. Following this notation we will use ${}_{E}^{S}q_{t}$ to refer to the current value of the quaternion that represents the orientation of frame *E* (inertial/Earth frame), relative to the frame *S* (sensor/local frame). Applying Madgwick’s method [29,30], we can obtain the quaternion that can be used to project the sensors readings obtained in the local frame, linked to the mobile, into the inertial reference system (Earth frame). In particular, we will use the simplest version of the algorithm, that obtains this quaternion by using the information provided by the gyroscope and the accelerometer sensors, without using the magnetometer data.

The gyroscope measures the angular velocity around the *x*, *y*, and *z* axes of the local system, termed $\omega_{x}$, $\omega_{y}$ and $\omega_{z}$ respectively. If these parameters are arranged into the ${}^{S}\omega$ vector defined by Equation (3), the quaternion derivative describing the rate of change of orientation of the Earth frame relative to the sensor frame (local frame), ${}_{E}^{S}\dot{q}$, can be calculated using Equation (4):

(3) $${}^{S}\omega =\left[0\;\omega_{x}\;\omega_{y}\;\omega_{z}\right],$$

(4) $${}_{E}^{S}\dot{q}=\frac{1}{2}{}_{E}^{S}q⊗{}^{S}\omega ,$$

where ⊗ is the quaternion product. Therefore, the orientation of the Earth frame relative to the local one at time *t*, ${}_{E}^{S}q_{t}$, can be computed by integrating over time the quaternion derivative:

(5) $${}_{E}^{S}q_{t}={}_{E}^{S}q_{t-1}+\left(\frac{1}{2}{}_{E}^{S}q_{t-1}⊗{}^{S}\omega_{t}\right)\times \Delta t,$$

where ${}^{S}\omega_{t}$ is the angular rate provided by the gyroscope and measured at time *t*, Δt is the sampling period, and ${}_{E}^{S}q_{t-1}$ and ${}_{E}^{S}q_{t}$ are the previous and current estimations of the quaternion ${}_{E}^{S}q$.

The gyroscope has a high rate error, its data drifts over time, is unstable, and low angular velocities might not be properly registered. Because of all this, and to compensate these errors, it is possible to use the accelerometer readings to correct the estimation of the quaternion. The accelerometer measures the accelerations experienced by the mobile in the three axes of the local reference system: $a_{x}$, $a_{y}$ and $a_{z}$. Once again, these values can be arranged into a four-dimensional vector, ${}^{S}a$:

(6) $${}^{S}a=\left[0\;a_{x}\;a_{y}\;a_{z}\right].$$

When the mobile is not subdued to any other external forces but the gravity, the projection of the unit gravity vector in the Earth frame, ${}^{E}g=\vec{G}\;/\;\left\|G\right\|=\left\{0,0,0,1\right\}$, into the local reference system (body frame), should coincide with the information detected by the tri-axial accelerometer ${}^{S}a=\left\{0,a_{x},a_{y},a_{z}\right\}$. This projection of ${}^{E}g$ into the local reference system can be computed as:

(7) $${}_{E}^{S}q_{t}^{*}⊗{}^{E}g⊗{}_{E}^{S}q_{t},$$

where ${}_{E}^{S}q_{t}^{*}$ is the conjugate of ${}_{E}^{S}q_{t}$. Hence, it should happen that ${}_{E}^{S}q^{*}⊗{}^{E}g⊗{}_{E}^{S}q={}^{S}a$. This is the reason why the quaternion will be the one that minimizes the difference shown in Equation (8):

(8) $${}_{E}^{S}q=\underset{{}_{E}^{S}q\in R^{4}}{\arg\;\min}\;\left({}_{E}^{S}q^{\;*}⊗{}^{E}g⊗{}_{E}^{S}q-{}^{S}a\right).$$

Finally, we can add up the two sources of information, Equations (5) and (8), to achieve the quaternion at every instant, as shown in Equation (9), where *f* comes from Equation (8), such that $f={}_{E}^{S}q^{\;*}⊗{}^{E}g⊗{}_{E}^{S}q-{}^{S}a$:

(9) $${}_{E}^{S}q_{t}=\;{}_{E}^{S}q_{t-1}+\gamma \left(-\mu \frac{\nabla f}{\left||\nabla f|\right|}\right)+\left(1-\gamma \right)\left(\frac{1}{2}{}_{E}^{S}q_{t-1}⊗{}^{S}\omega_{t}\right)\times \Delta t.$$

### 3.2. Estimation of the Acceleration in the Earth Frame

Once we know the attitude of the phone, Equation (9), we can now obtain the three components of the acceleration experienced by the mobile in the Earth reference system, ${}^{S}a_{E,t}$, which we will call *projected acceleration* henceforth:

(10) $${}^{S}a_{E,t}={}_{E}^{S}q_{t}⊗{}^{S}a_{t}⊗{}_{E}^{S}q_{t}^{*},$$

where ${}^{S}a_{t}$ is the vector that arranges the accelerometer readings (in the local frame) at time *t*.

### 3.3. Signal Filtering and Centering

At this stage, we have the following 9-dimensional time series $s_{t}$:

(11) $$s_{t}=\left[\begin{array}{c} a_{t}\\ a_{E,t}\\ \omega_{t} \end{array}\right],$$

where $a_{t}\in R^{3}$ is the acceleration data in the sensor frame, $a_{E,t}\in R^{3}$ is the projected acceleration data in the Earth frame that we have just introduced in Equation (10), and $\omega_{t}\in R^{3}$ is the angular velocity data in the sensor frame.

In Section 4 we will present several proposals for walking recognition that use as inputs some components of the above time series. However, we can still carry out two more preprocessing tasks which will help to improve the performance of our algorithms. These two task are a frequency domain filtering and a signal centering.

Since most of the energy captured by the accelerations and angular rates associated with human walking is bellow 3 Hz [31], we can apply a low-pass filter over the components in Equation (11) to minimize the signal noise. Specifically, we use a 10th order Butterworth filter with a 3 Hz cut-off frequency. In this way, we remove the high-frequency components of the noise.

The presence of a non-zero DC component can hide important information, especially in the frequency domain. To solve this issue, we apply a DC-bias filter to center the signal:

(12) $${\tilde{s}}_{t}=s_{t}-\frac{1}{N}\sum_{n=1}^{N}s_{t-n},$$

where the second term is the signal mean value computed using a moving average filter and *N* is the length of the moving average, in our case, N=250.

## 4. Walking Recognition

To identify when the user is walking, we addressed the problem in two different ways: feature-based and shape-based approaches. Our aim is to explore the various ways to solve the problem with each of these strategies and then compare them by extracting the most relevant advantages and disadvantages.

In the first case, the feature-based approach, the classifier is built from the most representative feature set, extracted from time-series data. In the case of the shape-based approach, the classifier uses directly the shape of the time series to detect characteristic patterns. In both cases, to determine whether the user is walking, the classifiers work with a window that comprises the last 250 sensor measurements: ${\tilde{s}}_{t},{\tilde{s}}_{t-1},\ldots ,{\tilde{s}}_{t-249}$, from Equation (12). Hence, we work with sliding windows of N=250 samples and 50% overlap. Since we sample at 100 Hz, 250 samples correspond to 2.5 seconds. As each new window overlaps with half of the previous one, we can make a new prediction (i.e., say whether the person is walking or not) every 1.25 seconds. We chose this way of partitioning based on previous results [19,32].

### 4.1. Feature-Based Classification

Following this approach, we use supervised learning to differ walking from non-walking sequences in the signal. In this case, the classifier starts from a set of features computed from the data window described in the previous section. This set of features, which can be either manually or automatically determined, should be meaningful and contain relevant information to identify the walking activity. Using feature vectors instead of raw data can reduce the number of input elements and improve the generalization ability. To obtain this feature set, we applied both manual feature selection techniques for traditional machine learning algorithms, as well as deep learning networks, which automatically extract the most relevant features from the data.

#### 4.1.1. Classification Methods Using Manual Feature Selection

To use traditional machine learning algorithms, we manually collected a total of 46 features both in temporal and frequency domains based on previous works [2,19,33,34,35,36], Then, we analyzed the relevance of each feature and discarded those redundant or irrelevant through the combined use of two feature selection techniques: Recursive Feature Elimination (RFE) [37] and Correlation-based Feature Selection (CFS) [38]. The final subset is made up of 21 variables.

The selected features in the time domain are:

- $E_{a_{E,z}}$: the energy of the vertical component of the projected acceleration;
- $E_{\omega }$: the energy of the gyroscope norm;
- $\sigma_{\omega }^{2}$: the variance of the gyroscope norm;
- $\sigma_{a_{x}}$, $\sigma_{a_{y}}$ and $\sigma_{a_{z}}$: the standard deviation for each axis of the acceleration;
- $\sigma_{a_{E,x}}$, $\sigma_{a_{E,y}}$ and $\sigma_{a_{E,z}}$: the standard deviation for each axis of the projected acceleration;
- $ZCR_{a}$: the zero-crossing rate of the acceleration norm;
- $P_{a}$: the peak count of the acceleration norm;
- $P_{a_{E,z}}$: the peak count of the vertical projected acceleration;
- $\varsigma_{a_{E,z}}$: the skewness of the vertical projected acceleration;
- $\varsigma_{\omega }$: the skewness of the gyroscope norm;
- $\kappa_{a_{E,z}}$: the kurtosis of the vertical projected acceleration.

The previous energy features ($E_{a_{E,z}}$ and $E_{\omega }$) are computed as:

(13) $$E_{a_{E,z}}=\frac{1}{N}\sum_{n=1}^{N}\left|a_{E,z,n}\right|,$$

(14) $$E_{\omega }=\frac{1}{N}\sum_{n=1}^{N}\omega_{n},$$

where *N* is the length of the window and $\omega_{n}$ is the norm of the gyroscope data at the temporal index *n* of the window:

(15) $$\omega_{n}=\|\omega_{n}\|=\sqrt{\omega_{x,n}^{2}+\omega_{y,n}^{2}+\omega_{z,n}^{2}}.$$

The zero-crossing rate for the acceleration norm is computed according to:

(16) $$ZCR_{a}=0.5\times \frac{1}{N}\sum_{n=1}^{N-1}\left|sign\left(a_{n+1}\right)-sign\left(a_{n}\right)\right|$$

where sign(x) is the sign function, which returns the sign of a real number:

(17) $$sign\left(x\right)=\left\{\begin{array}{cc} 1, & x>=0\\ 0, & x<0 \end{array}\right.$$

and $a_{n}$ is the norm of the acceleration data at the temporal index *n*:

(18) $$a_{n}=\|a_{n}\|=\sqrt{a_{x,n}^{2}+a_{y,n}^{2}+a_{z,n}^{2}}.$$

The peak count is just the number of the peaks identified in each sliding window as local maximum points. Finally, for estimating the skewness and kurtosis metrics, we followed the third method proposed by Joanes and Gill in [39].

Regarding the frequency domain, all the features were extracted from the frequency spectrum of the norm of the original acceleration, *f*, and the frequency spectrum of the vertical component of the projected acceleration, $f_{E}$. To obtain these spectrums, we applied the Fast Fourier Transform (FFT) over each sliding window. The relevant features extracted from the spectrums are:

- $\mu_{f_{E}}$: the mean frequency of the vertical component of the projected acceleration;
- $\sigma_{f_{E}}$: the standard deviation of the previous mean frequency;
- $Md_{f_{E}}$: the median frequency of the vertical projected acceleration;
- $Mo_{f_{E}}$: the modal frequency of the vertical projected acceleration;
- $Mo_{f}$: the modal frequency of the acceleration norm;
- $\kappa_{f_{E}}$: the kurtosis of the spectrum of the vertical projected acceleration.

We evaluated this feature set with several classifiers: Random Forests, Support Vector Machines (SVM), Gradient Boosting Machines (GBM), *k*-Nearest Neighbors (*k*NN), Naïve Bayes and C5.0. In Section 5 we describe the experimental results obtained.

#### 4.1.2. Deep Learning

We also explored deep learning for feature selection and modeling. In recent years, deep learning has made great progress in the field of human activity recognition [40,41]. Unlike traditional machine learning methods, such as those mentioned in Section 4.1.1, deep learning methods perform gait behavior features extraction in a supervised and automatic way and can significantly improve the accuracy of recognition.

We used some convolutional neural network (CNN) architectures to extract the walking characteristics of data which is collected from inertial sensors. CNNs are a kind of deep networks that often consists of an input and an output layer, as well as multiple hidden layers. The hidden layers are typically a series of convolutional layers that convolve with a multiplication or other dot product. The activation function is commonly a ReLU layer, and is subsequently followed by additional convolutions such as pooling layers, fully connected layers and normalization layers. CNNs are very powerful extracting abstract features, especially in the context of image recognition processing. Due to the outstanding ability of CNNs in image processing, many researchers used them for gait or activity recognition [40,42].

CNNs are by nature computationally expensive and this could be a problem, since we are focusing on exploring several ways of solving the task in the context of mobile devices, which have a modest hardware. Moreover, CNNs often stand out from other learning methods when there are huge amounts of data to feed them with, but such large amounts of data are not always available, as in our case. An overly complex CNN architecture will be too computationally expensive to integrate into a smartphone application and will lead to underfitting when the amount of data is limited. Therefore, as in this work we intend to propose feasible solutions, we designed the simplest possible architectures so that they can be used not only in these kind of devices providing good performance, but even trained using only their hardware. Hence, our networks have few layers and, consequently, few parameters to tune. Furthermore, we only use one dimension from the 9-dimensional time series ${\tilde{s}}_{t}$ –Equation (12)– as input layer. The best candidates are the vertical component of the projected acceleration, $a_{E,z}$ and the norm of the acceleration in the sensor frame, $\left\|a_{n}\right\|$ –Equation (18)–. These components, $a_{E,z}$ and $\left\|a_{n}\right\|$, are the ones that we consider the most representative of the walking activity. Thus, our CNN classifiers will automatically learn features from patterns made up of the last 250 values of one of these 1-dimensional signal inputs, which will allow them to classify new patterns never seen before. Sacrificing the rest of the dimensions and using only one of them allows us to work with smaller network topologies. Moreover, as we will see in Section 5, working with CNNs with a single-dimension input layer is sufficient to obtain high performance in our particular problem.

The details about the different network architectures that we explored as well as the performance that we obtained using each one are exposed in Section 5 of experimental results.

### 4.2. Shape-Based Classification

As we already mentioned in Section 2, another way of dealing with the classification problem is by directly analyzing the shape of acceleration time series. In this raw-data-based approach, the classifiers will label patterns made up of the last 250 values of the vertical component of the projected acceleration, $a_{E,z}:a_{E,z}\left(t\right),a_{E,z}\left(t-1\right),\ldots ,a_{E,z}\left(t-249\right)$. We could have used more than just one dimension of the time series, but this would increase the computational cost of this approach too much. Therefore, we selected the component with the most representative shape to solve the problem, i.e., the vertical component of the projected acceleration, $a_{E,z}$.

Nevertheless, working with these raw data is not straightforward, since this is a distance-based classification, in which most classifiers label the patterns by comparing them with some kind of prototypes (support vectors in the SVMs, the *k* nearest neighbors in *k*NN, etc.). The problem is that this comparison includes the use of some distance metric, being the most common the Euclidean distance. The Euclidean distance has been widely used since it provides a simple and mathematically convenient metric on raw data. However, this metric is not convenient for the problem being tackled, due to its sensitivity to distortion of the patterns in the time axis (two identical patterns might differ significantly by something as simple as the fact that both patterns are shifted one with respect to the other by only one sample). As we work with segments of time series, the best option to perform matching among these sequences is by a non-linear stretching and contracting of the time axes, using techniques, such as dynamic time warping (DTW) [24]. In fact, as it has been pointed out in [43,44], DTW provides an elastic matching of two sequences while Euclidean distance is too brittle since it only allows one-to-one point matching. The Euclidean distance is sensitive to offset, amplitude, scaling, noise, phase shifts and temporal distortion. On the contrary, DTW can be used to align time series in a non-linear manner by minimizing the distance among them. DTW allows local contraction and expansion of the time axis. Originally, this technique was used to compare different speech patterns in automatic speech recognition, determining if two waveforms represented the same spoken phrase [45]. In addition to speech recognition, DTW has also been found useful in many other disciplines [46], including data mining, gesture recognition, robotics, manufacturing and medicine.

DTW employs dynamic programming to compare and align two temporal sequences (that might even differ in length), trying to minimize the distance between the two of them [24]. Thus, if $x=(x_{1},\ldots ,x_{n})$ and $y=(y_{1},\ldots ,y_{m})$ are the two finite series taking values in a space χ, the alignment π between x and y, of length |π| is a pair of increasing *p*-tuples $\left(\pi_{1},\pi_{2}\right)$ such that:

(19) $$\begin{array}{c} 1=\pi_{1,1}\leq \ldots \leq \pi_{1,p}=n,\\ 1=\pi_{2,1}\leq \ldots \leq \pi_{2,p}=m. \end{array}$$

Hence, the distance among the two aligned time series (dynamic time warping distance) can be computed as:

(20) $$d_{DTW}\left(x,y\right)=P\left(\pi \right)=\sum_{i=1}^{\left|\pi \right|}\varphi \left(x_{\pi_{1,i}},y_{\pi_{2,i}}\right),$$

where φ is a cost, in our case the Euclidean distance. Dynamic programming algorithms provide an efficient way to compute the optimal path $\pi^{*}$ in terms of mean-score with respect to π:

$$\pi^{*}=\underset{\pi }{\arg\min}\frac{1}{\left|\pi \right|}P\left(\pi \right).$$

Although DTW is a robust distance metric, it is not always easy to integrate it into our distance-based classifiers. For example, in the particular case of a SVM, deriving a kernel to train the classifier based on the dynamic time warping distance is not a feasible solution, since in general DTW is not positive semi-definite (PSD) [43,47]. Any kernel function intended for the SVM must satisfy Mercer’s condition [48], i.e., it must be symmetric and positive semi-definite (PSD). Several ad-hoc strategies have been proposed for including non-PSD kernels in SVMs. The most immediate one is to simply ignore the fact that the kernel is non-PSD and see how it performs, but in this case the existence of a Reproducing Kernel Hilbert Space (RKHS) is not guaranteed [49] and it is no longer clear what is being optimized during the training of the SVM. Moreover, the resulting optimization problem may be non-convex, making it difficult to achieve a solution.

**Figure 2:**
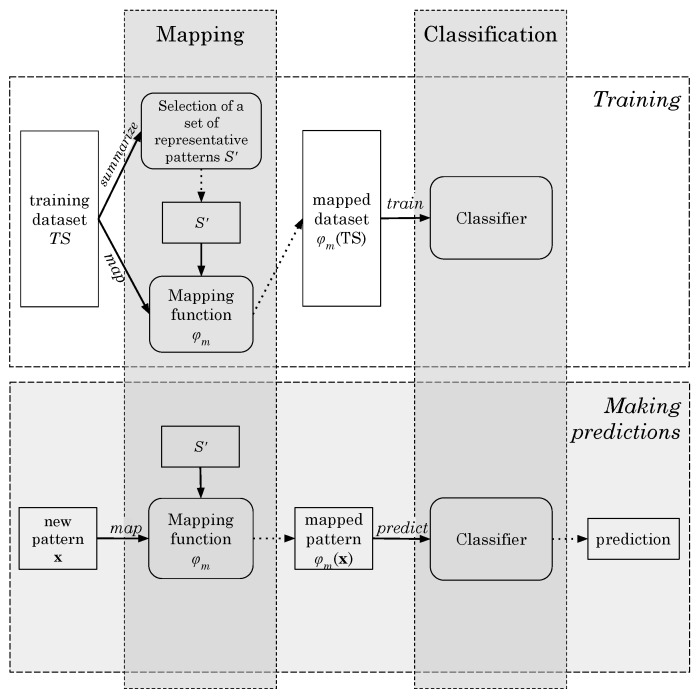
Workflow diagram of the shape-based proposal.

Hence, to work with DTW and distance-based classifiers, we suggest a solution inspired in what is know as *pairwise proximity function SVM* (ppfSVM) [43,50,51,52]. Our strategy operates in two stages: mapping and classification (Figure 2). Given a certain pattern x, the mapping stage projects it into a new space, $\phi_{m}\left(x\right)$, that reflects the distance (DTW) of x to several representative patterns:

(21) $$\phi_{m}:x\to \left(d_{DTW}\left(x,z_{1}\right),d_{DTW}\left(x,z_{2}\right),\ldots ,d_{DTW}\left(x,z_{m}\right)\right),$$

where $z_{i}$, with i=1,…,m is a set of representative patterns ($S^{\prime }$).

Therefore, given any pattern x, the classification proposed in this section is determined by $\phi_{m}\left(x\right)$, Equation (21), i.e., the distances among x and a set of representative patterns in $S^{\prime }$. For some distance-based classification methods, such as the *k*-Nearest Neighbors or the aforementioned ppfSVM, the set of representative patterns $S^{\prime }$ is the whole training dataset (TS) itself. Nevertheless, in our case, we cannot work with a big set $S^{\prime }$ since the mapping step $\phi_{m}\left(x\right)$ would take too long for the real time application of our classifier. Therefore, and as we will see later, $S^{\prime }$ is a reduced and small subset of the training data, i.e., $S^{\prime }\subset TS$.

**Figure 3:**
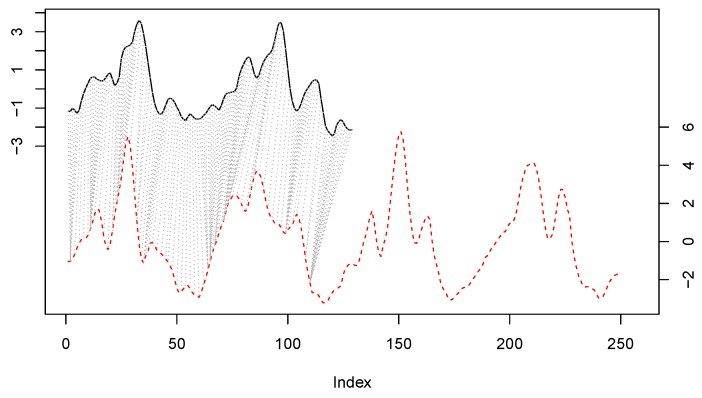
Subsequence DTW between a query representative pattern (the top black line) and another reference pattern x (the bottom red dotted line).

We still decided to carry out a further improvement. As we pointed out before, the input pattern x is a 250-dimensional vector that might contain the sequence of values ($a_{E,z}$) corresponding to several steps (if the user is walking). Because of this, we split the representative patterns of $S^{\prime }$ in half and we apply what is know as *subsequence DTW* (SDTW) [53]. Hence, any pattern $z\in S^{\prime }$ will now have 125 components. These 125 points might comprise the sequence of points corresponding to a bit more than one single step. In this case, performing SDTW between any x and any z is equivalent to searching whether the step represented by z is present in the pattern x. In this way, instead of aligning these sequences x and z globally, we will search the subsequence $z\in S^{\prime }$ within the longest patterns x. The subsequence DTW, also called “unconstrained” or open-begin-end (OBE-DTW), is achieved relaxing both the start-point and the end-point, discovering the contiguous part of the x pattern that best matches the whole half representative pattern z, which we will refer to, respectively, as the *reference* and *query* patterns from now on. Figure 3 shows graphically how this matching process is done. The small top signal is z (query), while the longest bottom signal is an instance x (reference). The figure shows how the best alignment is found in the first half of the reference.

Let $x=(x_{1},\ldots ,x_{n})$ and $z=(z_{1},\ldots ,z_{m})$ be the two vectors that we want to compare and align using subsequence DTW. The first one, x, is the reference, while z is the query. We assume that the length *n* is much larger than the length *m*. The goal of SDTW is to find a subsequence $x_{\left(a^{*}:b^{*}\right)}=\left(x_{a^{*}},\ldots ,x_{b^{*}}\right)$ with $1\leq a^{*}\leq b^{*}\leq n$ that minimizes the DTW distance to z over all possible subsequences of x:

(22) $$\left(a^{*},b^{*}\right)=\underset{(a,b):1\leq a\leq b\leq n}{\arg\min}\left(d_{DTW}\left(x_{\left(a:b\right)},z\right)\right).$$

Thus, we can define the subsequence dynamic time warping distance function as:

(23) $$d_{SDTW}=d_{DTW}\left(x_{\left(a^{*}:b^{*}\right)},z\right).$$

After all this process, the final mapping $\phi_{m}\left(x\right)$ that is used in our shape-based approach is:

(24) $$\begin{array}{cc} \phi_{m}:x\to & (d_{SDTW}\left(x,z_{1}\right),d_{SDTW}\left(x,z_{2}\right),\\ & \ldots ,d_{SDTW}\left(x,z_{m}\right)). \end{array}$$

For the second stage described in Figure 2, i.e., the classifier that labels the projected patterns $\phi_{m}\left(x\right)$, we used the same classifiers tested for the feature-based approach: Random Forests, Support Vector Machines (SVM), Gradient Boosting Machines (GBM), *k*-Nearest Neighbors (*k*NN), Naïve Bayes and C5.0. Please note that the first stage of our strategy, the mapping, allows us not only to use distance-based classifiers in the second stage (such as SVM or *k*NN), but also any other classifier, including statistical algorithms, rule-based methods, neural networks, etc. In Section 5 we will show the results that we obtained.

Thus, summarizing what has been described so far, we need a set of representative patterns (queries) $S^{\prime }$, included in the training set ($S^{\prime }\in TS$), with which perform the mapping described in Equation (24). Hence, given any pattern x (reference), we will classify $\phi_{m}\left(x\right)$ as walking or not walking. To obtain $S^{\prime }$ from TS we explored different strategies, in particular when $S^{\prime }$ is made up of:

- support vectors of an SVM trained using TS as training set (Section 4.2.1),
- medoids obtained after using a clustering algorithm (PAM), over the original training data TS (Section 4.2.2), and
- most representative patterns found through a supervised summarization procedure (Section 4.2.3).

#### 4.2.1. Support Vectors of a SVM as Representative Patterns

The first option we analyzed is to train a standard SVM using the original training set TS and the Euclidean distance to compare the temporal sequences. According to what has been pointed out before, we know that this solution is not appropriate to solve the classification problem, but here we are only interested in the support vectors achieved after the training stage, i.e., we take the resulting *m* support vectors of the SVM after the training, as the new set $S^{\prime }$. We proceed in this way, as the support vectors are the training examples that lie in the optimal frontier, i.e., the hyperplane that maximizes the margin between the two classes when the Euclidean distance is being used.

#### 4.2.2. PAM Medoids as Representative Patterns

The second option we explored to summarize the original training data TS into a reduced set $S^{\prime }$, is the application of a clustering technique, *k*-medoids [54]. *k*-medoids is a clustering method related to *k*-means in the sense that its objective is to partition the data into *k* sets, where each cluster is represented by the most centrally located object in the cluster, called *medoid*.

The most popular heuristic algorithm used to find good medoids is the *partitioning around medoids* (PAM) [54]. The PAM algorithm has two phases: (1) *Build*, in which a collection of *k* patterns are selected for the initial set $S^{\prime }$. (2) *Swap*, which tries to improve the quality of the clustering by exchanging those patterns selected as medoids in $S^{\prime }$ with the unselected ones. The pseudo-code can be found in Algorithm 1.
**Algorithm 1:** Partitioning Around Medoids (PAM).
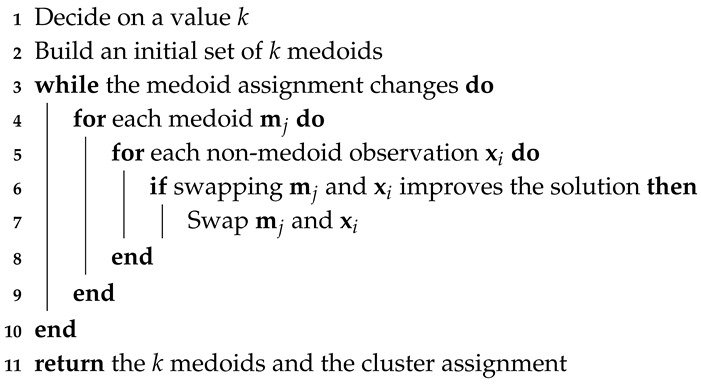


After applying Algorithm 1, the medoid of any cluster *j* fulfills the following condition:

(25) $$m_{j}=\underset{x_{i},\;{\tilde{x}}_{l}\;\in \;C_{j}j}{\arg\min}\sum d_{SDTW}\left(x_{i},{\tilde{x}}_{l}\right),$$

where $m_{j}$ represents the medoid of the cluster *j*, $C_{j}$, and $x_{i}$ and $x_{l}$ are patterns included in that cluster. It is important to realize that in the previous Equation (25) we wrote ${\tilde{x}}_{l}$ since, as we explained before, we use subsequence DTW and the query is made by a pattern, in this case $x_{l}$, divided in half, which we represent with a tilde. We must be aware of the fact that the *k* medoids achieved after the application of Algorithm 1 are patterns that belong to the training data TS, and which will now make up the set $S^{\prime }$ used in the mapping function $\phi_{m}$ from Equation (24).

Medoids are quite useful for data summarization because they are the most prototypical elements of their respective clusters. A big advantage of *k*-medoids over other partitive clustering algorithms like *k*-means is that PAM is more robust in the presence of noise and outliers, as well as the fact that any distance metric (DTW in our case) can be used. Moreover, *k*-medoids explores a smaller solution space than *k*-means. However, a potential disadvantage of this summarization with *k*-medoids or any other conventional clustering algorithm is that it is applied in a completely unsupervised way, using an error function that is only based on inter-pattern distances. It does not take into account the labels of the patterns. Because of this, we investigated the third method, “supervised summarization”, described in the next section.

#### 4.2.3. Supervised Summarization

To get $S^{\prime }$ from TS, we explored this third method which is inspired on a data mining technique called *supervised summary generation* (SSG) [55]. The objective of this *supervised summarization* is the creation of class-centered summaries that represent patterns that are typical for a class. In our case, the supervised summarization we applied generates a hard partition of the space in regions, so that each region contains mostly patterns belonging to the same class (Algorithm 2).
**Algorithm 2:** Supervised summarization.
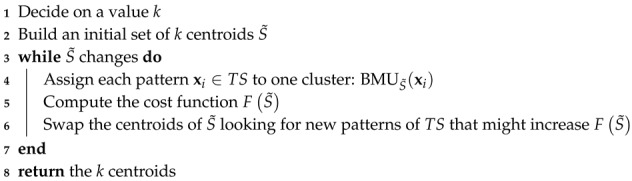


Thus, if we have an initial set of *k* centroids $\tilde{S}\subset TS$, given any input pattern x we use a winner-takes-all strategy to assign this pattern to only one cluster:

(26) $$BMU_{\tilde{S}}\left(x_{i}\right)=\underset{\forall {\tilde{x}}_{l}\;\in \;\tilde{S}}{\arg\min}\;d_{SDTW}\left(x_{i},{\tilde{x}}_{l}\right).$$

Then, we define a cost function $F(\tilde{S})$, which reflects to what extent the clusters are homogeneous considering the classes of the patterns included in each of them:

(27) $$F(\tilde{S})=\sum_{i=1}^{n}\;\delta \left(\mathrm{Class}\left(x_{i}\right)=\mathrm{Class}\left(BMU_{\tilde{S}}\left(x_{i}\right)\right)\right),$$

where $\tilde{S}\subset TS$ is the current subset of *k* centroids (representative patterns), $x_{i}$ the *i*-th pattern in TS, *n* is the cardinal of TS and Class(x) is the class of a given pattern x.

Thus, the higher the values of $F(\tilde{S})$, the better the solution represented by this set $\tilde{S}$. Therefore, we will iterate the patterns in $\tilde{S}$ looking for those which optimize $F(\tilde{S})$:

(28) $$S^{\prime }=\underset{\tilde{S}\;\subset \;TS}{\arg\max}\;F(\tilde{S}).$$

Hence, $S^{\prime }$ represents the subset of TS with the *k* representative patterns that maximizes *F*.

The swapping of the elements in $\tilde{S}$ to look for new candidates with which improve *F* (Algorithm 2) is not trivial. If *k* is very small a brute-force search is possible, but it will not be feasible if either the cardinal of the TS or the number of centroids, *k*, increases. In our case, for values of *k* higher than 2, we applied widely known heuristic methods common in optimization problems in order to perform an informed search and find quasi-optimal summaries of a greater number of patterns. In particular, we used *breadth-first search* (BFS) [56] and *simulated annealing* (SA) [57,58] methods.

## 5. Experimental Analysis and Results

We analyzed the performance of all the different proposals for walking recognition described in this paper: feature-based and shape-based classifiers, including the use of support vectors, PAM medoids or supervised summarization to get the mapping function described in the shape-based approach. To perform a fair comparison of these proposals, we first obtained a set of labeled data (ground truth), the description of which is included in Section 5.1. Finally, Section 5.2 shows the results obtained.

### 5.1. Ground Truth

To evaluate the performance of our proposals, we built a large dataset composed of a total of 140 records carried out by 77 different people. The vast majority of them (72, specifically) were volunteers not connected to the research, because we wanted to ensure that the data were not biased. In this way, in each record, the participant walked under natural conditions, freely or following some basic premises, while the information provided by the inertial sensors of the mobile phone being carried by the user was recorded and processed. Each volunteer walked, on average, about 2 min, giving around 110 steps.

We did an important effort to collect data in a wide variety of cases or situations. With this aim, we tried to get as many different volunteers as possible. On the other hand, when they moved, as they did it freely and in different environments, their speed and way of movement were different (walking in corridors, going upstairs, etc.), as it was the position of the mobile they carried (in their hand, pocket, backpack, etc.).

Obviously, one option to label these data, i.e., the steps walked by each user, would be manual counting. Nevertheless, this is error prone, especially if we consider the amount of data collected. There are commercial step counting solutions that perform particularly well when the user walks ideally (with the mobile in the palm of his/her hand) but which are susceptible to false positives when the movement or position of the mobile is far from the ideal one [59]. There are also some other solutions, described in medical literature, but which we have not used since they involve sensorized environments that constrain the freedom of movement of the user [60]. Besides, we want to emphasize that we are interested on an individual labeling of each step in the signal. Most articles in the bibliography evaluate the performance of their algorithms taking only into account the total number of steps detected per experiment or the total distance walked, instead of a detailed prediction about when the person is really walking and when is not [61,62,63]. We want to evaluate classifiers that distinguish whether each segment of signal corresponds to an user walking or not. This real time labeling of each segment of data will allow a fair analysis of the performance, avoiding the hiding effect due to the cancelling between false negatives and false positives.

**Figure 4:**
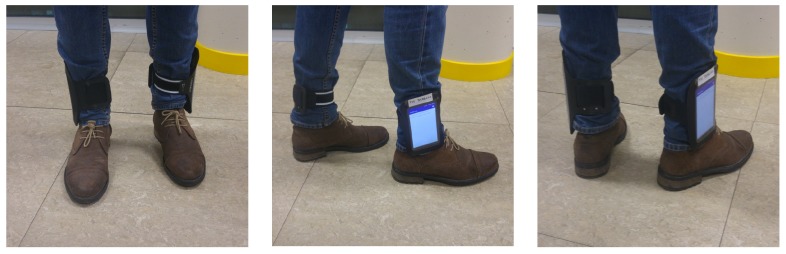
Sports armbands holding the mobiles of the legs.

Because all of this, and to achieve a reliable labeling of the inertial sensor signals, we decided that the volunteers had to carry a set of sensorized devices in their legs. In particular, they carried two other smartphones, one on each leg, tied with sport armbands, as shown in Figure 4. The inertial information registered in the legs is good enough to perform real time labeling and disambiguate when the user is really walking.

**Figure 5:**
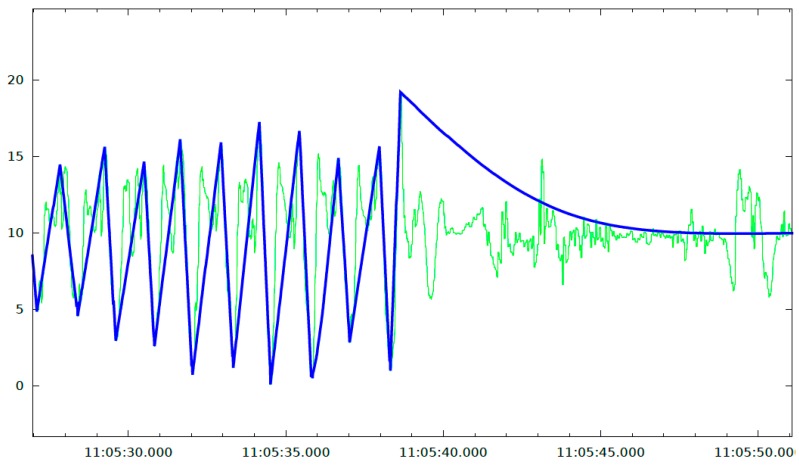
Graphical representation of the ground truth (thicker and darker line) over the signal of the vertical component of acceleration in the phone (thinner and clearer line).

Figure 5 shows a graphic representation of the ground truth over the signal of the vertical component of the acceleration registered in the main mobile phone. Each peak-valley sequence in the ground truth signal is equivalent to one step, so it is easy to identify when the user is really walking and when the phone is experiencing accelerations due to actions different from walking.

Our system of obtaining the ground truth fulfills its objective. However, given the large number of records performed with very varied characteristics, there will be situations in which the analysis of the signals for the identification of the steps is very complicated, even performing a manual count. In 28% of the records of the dataset, the ground truth has also been obtained by counting the steps manually, which allowed us to limit the error committed bellow 2%, i.e., between 1 and 2 steps per record.

We believe that our dataset could be useful for the community. For this reason, we published it online for anyone who wants to use it in their research. It can be consulted and downloaded at the following URL: https://citius.usc.es/investigacion/datasets/walking-recognition-dataset.

### 5.2. Performance Analysis

Now we are going to describe the evaluation and results obtained when each of our proposals, feature-based and shape-based ones, were tested over the dataset that we just described. All data preprocessing stages, as well as the training and testing of the different models, were carried out using the framework provided by the R language. In particular, for the training of the traditional models (Random Forests, SVM, *k*NN, etc.) we used the implementations already provided by the caret package [64]. In the case of the CNNs, we employed the keras package [65].

We split the records into sliding windows. Each window was labeled with the majority class of its samples: *walking* (positive) or *not walking* (negative). A problem with the division in sliding windows is that some of them coincide with moments in which the person carrying the mobile phone just stops or resumes walking. These windows are difficult to label because both activities occur in them, so they are noisy and can blur the results. Therefore, we kept only those windows in which at least 75% of the samples belong to the same class. After this, we had a total of 7886 labeled patterns. Nevertheless, this data was very unbalanced, because there were many more instances of the positive class, walking (78.1%) than of the negative class, not walking (21.9%). The imbalance in the training set can lead to bad models, because classifiers such as Random Forests are sensitive to the class distribution, i.e., they will tend to recognize better the class with the highest proportion of observations (known as the majority class). Therefore, to take advantage of all the data collected, we decided to work with an always balanced training set and leave the rest of the data for testing. Thus, the training set comprises 70% of the negative samples (1211 patterns) and the same number of positive patterns. Our training set, therefore, consisted of 2422 instances, which is 30.7% of the collected data. The remaining 69.3% was used for testing. This test set is used in this section of experimental results to make an evaluation of the different approaches always on the same data.

#### 5.2.1. Feature-Based Classification

We evaluated the feature-based proposal with several classifiers. Optimal hyperparameters for each classifier were estimated applying 10-fold cross validation on the training set. For each classifier we obtained its confusion matrix: true positives (TP), false positives (FP), true negatives (TN) and false negatives (FN). Then, we calculated the accuracy, sensitivity and specificity metrics.

**Figure 6:**
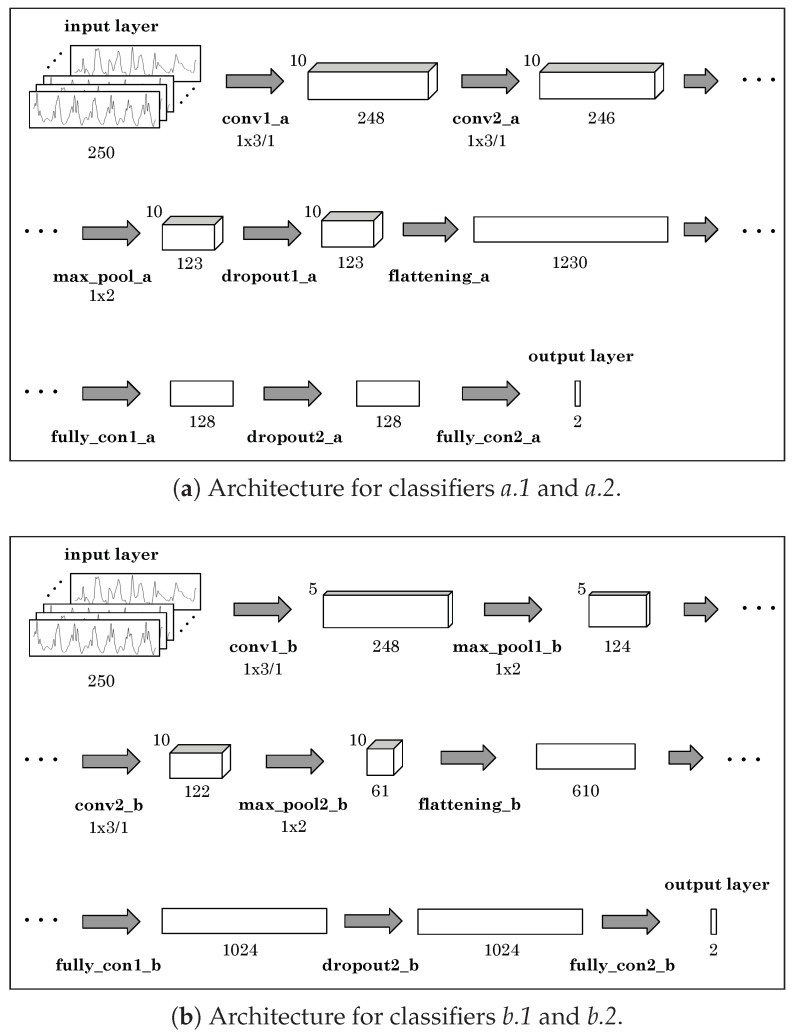
Graphical representation of the two different CNN architectures used in the experiments.

For deep learning, we tried two different CNN architectures. Considering that the input signal is one-dimensional, several one-dimension kernels are used in the convolution operations in the proposed networks. Figure 6 shows both architectures. The first one, shown in Figure 6a –architecture *a*–, is constructed with two convolutional layers, one max-pooling layer, two dropout layers, one flattening layer and two fully connected layers. The second one, shown in Figure 6b –architecture *b*– is a little more complex, constructed with two convolutional layers, two max-pooling layers, one flattening layer, one dropout layer and two fully connected layers. The total number of parameters for architecture *a* is 158176, and for architecture *b* is 627894. Table 1 and Table 2 show the details of architectures *a* and *b*, respectively. We evaluated both architectures using as input layer the vertical component of the projected acceleration, $a_{E,z}$ (classifiers *a.1* and *b.1*), and the norm of the acceleration in the device frame, $\left\|a_{n}\right\|$ (classifiers *a.2* and *b.2*). All CNNs were trained during 64 epochs using a batch size of 128 instances. A dropout rate of 0.2 was used in all the dropout layers.

Table 3 summarizes the results for all the classifiers tested, both traditional and those of deep learning. As we can see, the seven traditional classifiers provide very good results, although there are nuances. Some of them, such as *k*-Nearest Neighbors (*k*NN) and Naïve Bayes, despite providing competitive accuracies, present an imbalance between false positives and false negatives. The false positive rate (FPR=1−Specificity) is higher than the false negative rate (FNR=1−Sensitivity) in these cases. Random Forests provides the best results, followed closely by the radial basis function (RBF) kernel SVM. The percentage of false positives and false negatives is balanced in both cases. Using Random Forests only 19 of 519 negative instances are misclassified, which is equivalent to 3.66%, while 237 of 4945 positive instances are wrong, which is 4.79%. Random Forests is an ensemble of decision trees. In our case, the optimal number of trees was 400 and the minimum size of each terminal node was restricted to 1. The effectiveness of tree-based methods for activity recognition has been shown previously [19,66]. Random decision forests correct the tendency of decision trees of overfitting the training set. Therefore, we believe that Random Forests is the best traditional classifier for our feature-based proposal.

Regarding the CNNs, their results are slightly below those obtained using traditional methods. However, it must be borne in mind that this kind of learning techniques requires huge amounts of training data to perform well, much more than we have used so far. Since continuing to record more and more data was neither feasible nor honest for the comparison of classifiers, we decided to carry out an oversampling process to generate more instances artificially. First, we increased the overlap between consecutive data windows from 50% to 75%. Moreover, we upsampled the minority class (negative examples) using random resampling without replacement. Then, we kept 70% of the data for training and the remaining 30% for testing. This way, the deep learning training set increased from the original 2422 examples to 18,618, while the test set grew from 5464 to 6206 patterns.

When re-training the CNNs using the upsampled data, results improve significantly. Any of them outperforms the traditional methods. Comparing the four networks, we can affirm that both architectures, *a* and *b*, provide similar results, but architecture *a* is more interesting because it is the simplest in terms of number of parameters and, hence, it is the architecture that most likely exhibits the best generalization for new patterns. Similarly, both architectures are able to extract relevant features using any of the proposed signals as input layer. It could be more interesting to use the norm of the acceleration in the device frame, $\left\|a_{n}\right\|$ (classifiers *a.2* and *b.2*) because this avoids the preprocessing stage of quaternion calculation for attitude estimation. Thus, although training a CNN is computationally expensive, using these models would be more efficient than using traditional proposals once the model has been trained, since making predictions is inexpensive and they do not require some of the preprocessing stages. In conclusion, the most efficient deep learning network for our feature-based proposal would be the CNN *a.2*.

#### 5.2.2. Shape-Based Classification

We also evaluated the performance of the shape-based approach. As was described in Section 4.2, this strategy works in two stages: First, it defines a mapping that requires the selection of a set of *k* representative patterns of the training data. On a second stage, a classifier is trained using this new mapped space. To analyze this approach, we used different values for *k*, as well as the three strategies described in Section 4.2 to obtain the set of representative patterns $S^{\prime }$: the support vectors of an RBF SVM, *k* medoids of PAM and the outcome of a supervised summarization. For the calculation of the DTW distances, we used subsequence matching (SDTW) with asymmetric step pattern [67]. Finally, to perform the classification we used seven alternatives, the same seven classifiers dealt with in the previous section. Table 4 shows the results.

When we used the RBF SVM to obtain the set of representative patterns $S^{\prime }$, we obtained a total of 1551 support vectors. If we use all of them we get results very similar to those obtained via feature-based classification. If the final classifier is also another RBF SVM, the accuracy is 95.35%, being this performance a bit lower when we use a linear SVM instead. However, the computational cost of this approach grows excessively due to the number of representative patterns $S^{\prime }$ used to perform the mapping. Hence, we decided to reduce the cardinal of $S^{\prime }$ by applying feature selection techniques. First, due to the high dimensionality, we applied a filter method, because it is the most agile option: we ranked the features based on the Information Gain (IG) and Chi-squared ($\chi^{2}$) metrics [68] and we selected the first 221 features with the best scores (IG >0.3 and $\chi^{2}>0.7$). Then, we applied Recursive Feature Elimination (RFE) [37], which is a wrapper method, on that subset. RFE did not find any smaller subset with better performance, but with only the top 5 features we obtained quite good results. We want to emphasize that the specificity decreases significantly for the best models, by approximately 20%, or what is the same, false positive rate increases by 20%. Despite all, the false negative rate and the accuracy obtained are still quite good.

Using PAM as a selection strategy to obtain the set of representative patterns $S^{\prime }$, we can obtain good results too. As shown in Table 4, the results are quite competitive, taking into account the small number of patterns used. There are some global clustering quality indexes (Calinski-Harabasz index [69], C-index [70], Gamma-index or or Goodman-Kruskal index [71], Silhouette index [72], Gap Statistic [73], etc.) that can be used to automatically determine the optimal number of medoids. In our case, we calculate the Silhouette index, which is based on compactness and separation of clusters. We observed that low values of *k* (k=2) are sufficient to obtain good clustering results.

Finally, using supervised summarization the results are in the same range of values. First, we performed an exhaustive search in order to find the two best representative patterns. Then, due to computational limitations, we performed several informed searches using breadth-first search (BFS) and simulated annealing (SA) heuristic methods in order to find good subsets of four and ten patterns.

As we can see, the shape-based approach easily achieves accuracies above 90% whatever the configuration used. Nevertheless, globally, the shape-based approach is overtaken by the feature-based one. In particular, deep learning methods provide results that are difficult to achieve by more traditional strategies and they do not require a high effort in data preprocessing.

#### 5.2.3. Combination of Classifiers

As a final experiment, being aware that CNNs are the alternative that provides better performance, we tried to combine the predictions of the traditional feature-based Random Forests and the shape-based RBF SVM classifiers in order to see if, together, they are able to rival deep learning. For this purpose, we used two ensemble techniques.

First, we built a *weighted average* (WA) ensemble [74]. We applied 10-fold cross validation using training data and we calculated and saved the probabilistic predictions from each of the 10 folds for both classifiers. These values define the probability of walking in the range [0,1]. Then, we prepared a new dataset combining the predicted probabilities for each instance of the training set for Random Forests and RBF SVM (2 features) plus the real label. We applied logistic regression on the new dataset and obtained two coefficients derived from the logistic regression. We calculated linear weights based on the coefficients:

(29) $$w_{RF}=\frac{|c_{RF}|}{|c_{RF}\left|+\right|c_{SVM}|},$$

(30) $$w_{SVM}=\frac{|c_{SVM}|}{|c_{RF}\left|+\right|c_{SVM}|},$$

where $w_{RF}$ and $c_{RF}$ are, respectively, the weight and the coefficient for the Random Forests and $w_{SVM}$ and $c_{SVM}$ the weight and the coefficient for the SVM. In our case, we obtained $w_{RF}=0.9721$ and $w_{SVM}=0.0278$. Finally, we calculated the ensemble learning prediction probability score, $p_{e}$, by multiplying weights with predicted scores of each classifier:

(31) $$p_{e}=w_{RF}\times p_{RF}+w_{SVM}\times p_{SVM},$$

being $p_{RF}$ the prediction given by the feature-based Random Forests model and $p_{SVM}$ the prediction given by the shape-based RBF SVM model. Discretizing the probabilistic prediction $p_{e}$, we obtained the results of Table 5, which are exactly the same as we got for Random Forests in Table 3. This is because the logistic regression assigns a very high weight to the Random Forests and the prediction of the SVM practically does not influence the results. Therefore, in our case, using a weighted average model does not bring any benefit.

The second approach we tried was *stacking* [75]. We can train a new model using the same dataset that we used to apply logistic regression, composed by the predicted probabilities of the feature-based Random Forests and the shape-based RBF SVM classifiers as independent variables and the original target variable as dependent variable. In this case, the trained model is called *top layer model*. For each new prediction, we have first to obtain the predicted probabilities of the bottom layer models and then obtain the final prediction using the top layer one. We evaluated 7 different models in the top layer. The results are shown in Table 5. As we can see, we obtained slight improvements using another RBF SVM or C5.0 in the top layer. However, it is not too significant to make it worthwhile to use this new layer in a real application and it still does not improve the results of Table 3 obtained using the CNNs.

## 6. Conclusions

The use of the inertial information in mobile phones to recognize when a person is walking is an important issue in tasks such as biometric identification, indoor navigation, health monitoring, etc. Nevertheless, walking recognition in mobile phones is challenging due to the existence of a high aliasing, i.e., we get very similar signals for many different movements of the mobile. In this paper, we reviewed the state of the art in this field and we carried out an exhaustive analysis and development of two different approaches to detect walking activity on a person carrying a mobile device. Both of them process the information provided by the inertial sensors of the device to make continuous predictions. The first one, feature-based classification, is based on extracting features of time and frequency domains from the accelerometer and gyroscope readings. The second one, shape-based classification, focuses on comparing the shape of the vertical component of the acceleration (in the Earth reference system) with representative (query) patterns.

Using the feature-based classification, the best model has proved to be a simple CNN that uses the norm of the acceleration in the mobile frame as input layer. In any case, if we have limited resources, a traditional Random Forests is a competitive alternative when an appropriated manual feature selection is carried out. In the shape-based approach, we need to apply first a strategy able to determine representative patterns (queries) to which compare any input pattern. In fact, they will be the warped distances (DTW) among any input patterns and these queries what will later allow classifying the signal and, thus, recognize when the user is walking. We explored three different strategies to build the set of representative patterns (queries) as well as different strategies for classification. In general, we noticed that there are many combinations that provide good results, but they cannot compete with those achieved using deep learning. Finally, we also tried to combine traditional methods from feature-based and shape-based approaches, thus creating a small ensemble. We applied two different strategies, weighted average and stacking. However, the improvement obtained is not too significant to make worthwhile to use an ensemble in a real-time scenario.

Some further tests could be carried out in an attempt to improve the performance of the shape-based classifiers even further, for example using a different distance metric, such as longest common subsequence (LCSS), instead of DTW. Nevertheless, the expected benefits would be marginal considering the results already achieved. The evident conclusion of this work is that deep learning methods (in this case, CNNs) far outperform traditional learning methods. This work is a further proof of the advantages that deep learning can offer. In our problem, a small CNN architecture managed to simplify the data preprocessing stage by offering the highest accuracies. A priori, the main advantage of traditional methods is that they require much less data for the training of the model. In addition, they seem to be computationally lighter and, therefore, more appropriate for training and predicting in real time on smartphones. However, a deeper study should be done comparing traditional and deep learning methods running in a real mobile phone to test computational requirements and battery consumption.

## Figures and Tables

**Table 1:** Details of the CNN architecture *a*.

Layer Name	Kernel Size	# Kernels	Stride	Feature Map.	# Params
conv1_a	1 × 3	10	1	1 × 248 × 10	40
conv2_a	1 × 3	10	1	1 × 246 × 10	310
max_pool_a	1 × 2	-	1	1 × 123 × 10	0
dropout1_a	-	-	-	1 × 123 × 10	0
flattening_a	-	-	-	1 × 1230 × 1	0
fully_con1_a	-	-	-	1 × 128 × 1	157,568
dropout2_a	-	-	-	1 × 128 × 1	0
fully_con2_a	-	-	-	1 × 2 × 1	258

**Table 2:** Details of the CNN architecture *b*.

Layer Name	Kernel Size	# Kernels	Stride	Feature Map.	# Params
conv1_b	1 × 3	5	1	1 × 248 × 5	20
max_pool1_b	1 × 2	-	1	1 × 124 × 5	0
conv2_b	1 × 3	10	1	1 × 122 × 10	160
max_pool2_b	1 × 2	-	-	1 × 61 × 10	0
flattening_b	-	-	-	1 × 610 × 1	0
fully_con1_b	-	-	-	1 × 1024 × 1	625,664
dropout_b	-	-	-	1 × 1024 × 1	0
fully_con2 _b	-	-	-	1 × 2 × 1	2050

**Table 3:** Summary of results using the feature-based proposal with different classifiers.

Feature Selection Method	Classifier	TP	FP	TN	FN	Sensitivity	Specificity	Accuracy
Manual selection	Random Forests	4708	19	500	237	0.9521	0.9634	0.9531
	RBF SVM	4703	19	500	242	0.9511	0.9634	0.9522
	GBM	4707	29	490	238	0.9519	0.9441	0.9511
	*k*NN (k=5)	4723	48	471	222	0.9551	0.9075	0.9506
	Linear SVM	4642	44	475	303	0.9387	0.9152	0.9365
	Naïve Bayes	4654	61	458	291	0.9412	0.8825	0.9356
	C5.0	4633	48	471	312	0.9369	0.9075	0.9341
Deep learning	CNN (architecture *a.1*)	4632	38	481	313	0.9359	0.9282	0.9357
	CNN (architecture *a.2*)	4563	50	469	382	0.9210	0.9115	0.9210
	CNN (architecture *b.1*)	4567	32	487	378	0.9251	0.9211	0.9250
	CNN (architecture *b.2*)	4596	47	472	349	0.9276	0.9247	0.9275
Deep learning (oversampling data)	CNN (architecture *a.1*)	3100	3	3003	100	0.9834	0.9819	0.9834
	CNN (architecture *a.2*)	3080	17	3005	92	0.9824	0.9803	0.9824
	CNN (architecture *b.1*)	3098	5	3019	84	0.9857	0.9853	0.9857
	CNN (architecture *b.2*)	3069	28	3016	81	0.9824	0.9830	0.9824

**Table 4:** Summary of results using the shape-based proposal with different classifiers.

Pattern Selection Method	Classifier	No. of Patterns	TP	FP	TN	FN	Sensitivity	Specificity	Accuracy
RBF SVM support vectors	RBF SVM	1551	4724	33	486	221	0.9553	0.9364	0.9535
		221	4611	121	398	334	0.9325	0.7669	0.9167
		5	4586	126	393	359	0.9274	0.7572	0.9112
	Random Forests	1551	4674	35	484	271	0.9452	0.9326	0.9440
		221	4573	75	444	372	0.9248	0.8555	0.9182
		5	4378	109	410	567	0.8853	0.7900	0.8763
	GBM	1551	4668	37	482	277	0.9440	0.9287	0.9425
		221	4547	82	437	398	0.9195	0.8420	0.9122
		5	4497	116	403	448	0.9094	0.7765	0.8968
	Linear SVM	1551	4621	37	482	324	0.9345	0.9287	0.9339
		221	4449	52	467	496	0.8997	0.8998	0.8997
		5	4425	115	404	520	0.8948	0.7784	0.8838
	*k*NN (k=7)	1551	4703	69	450	242	0.9511	0.8671	0.9431
		221	4563	68	41	382	0.9228	0.8690	0.9176
		5	4330	105	414	615	0.8756	0.7977	0.8682
	Naïve Bayes	1551	4660	146	373	285	0.9424	0.7187	0.9211
		221	4607	132	387	338	0.9316	0.7457	0.9140
		5	4541	123	396	404	0.9183	0.7630	0.9036
	C5.0	1551	4400	56	463	545	0.8898	0.8921	0.8900
		221	4176	84	435	769	0.8445	0.8382	0.9439
		5	4683	136	383	262	0.9470	0.7380	0.9272
PAM medoids	RBF SVM	180	4651	47	472	294	0.9405	0.9094	0.9376
		10	4555	81	438	390	0.9211	0.8439	0.9138
		4	4623	104	415	322	0.9349	0.7996	0.9220
		2	4323	149	370	622	0.8742	0.7129	0.8589
	Random Forests	180	4633	57	462	312	0.9369	0.8902	0.9325
		10	4513	77	442	432	0.9126	0.8516	0.9068
		4	4410	91	428	535	0.8918	0.8247	0.8854
		2	3973	126	393	972	0.8034	0.7572	0.7990
	GBM	180	4598	53	466	347	0.9298	0.8979	0.9268
		10	4468	70	449	447	0.9035	0.8651	0.8999
		4	4500	94	425	445	0.9100	0.8189	0.9014
		2	4229	120	399	716	0.8552	0.7688	0.8470
	Linear SVM	180	4511	38	481	434	0.9122	0.9268	0.9136
		10	4544	89	430	401	0.9189	0.8285	0.9103
		4	4496	123	396	449	0.9092	0.7630	0.8953
		2	4311	156	363	634	0.8718	0.6994	0.8554
	*k*NN (k=7)	180	4629	66	453	316	0.9361	0.8728	0.9301
		10	4572	97	422	373	0.9246	0.8131	0.9140
		4	4434	92	425	445	0.9100	0.8189	0.9014
		2	4117	120	399	828	0.8326	0.7688	0.8265
	Naïve Bayes	180	4526	113	406	419	0.9153	0.7823	0.9026
		10	4346	79	440	599	0.8789	0.8478	0.8759
		4	4395	85	434	550	0.8888	0.8362	0.8838
		2	4172	156	363	773	0.8437	0.6994	0.8300
	C5.0	180	4362	76	443	583	0.8821	0.8536	0.8794
		10	4293	77	442	652	0.8681	0.8516	0.8666
		4	4593	109	410	352	0.9288	0.7900	0.9156
		2	4200	144	375	745	0.8493	0.7225	0.8373
Exhaustive search	RBF SVM	2	4492	93	426	453	0.9084	0.8208	0.9001
	Random Forests	2	4179	89	430	766	0.8451	0.8285	0.8435
	GBM	2	4306	78	441	639	0.8708	0.8497	0.8688
	Linear SVM	2	4360	85	434	585	0.8817	0.8362	0.8774
	*k*NN (k=7)	2	4293	91	428	625	0.8681	0.8247	0.8640
	Naïve Bayes	2	4135	91	428	810	0.8362	0.8247	0.8351
	C5.0	2	4587	120	399	358	0.9276	0.7688	0.9125
Informed search: Breadth-first search	RBF SVM	4	4526	68	451	419	0.9153	0.8690	0.9109
		10	4504	63	456	441	0.9108	0.8786	0.9078
	Random Forests	4	4441	68	451	504	0.8981	0.8690	0.8953
		10	4494	60	459	451	0.9088	0.8844	0.9065
	GBM	4	4411	62	457	534	0.8920	0.8805	0.8909
		10	4465	64	455	480	0.9029	0.8767	0.9004
	Linear SVM	4	4376	66	453	569	0.8849	0.8728	0.8838
		10	4443	69	450	502	0.8985	0.8671	0.8955
	*k*NN (k=7)	4	4434	75	444	511	0.8967	0.8555	0.8928
		10	4430	74	445	515	0.8959	0.8574	0.8922
	Naïve Bayes	4	4623	130	389	322	0.9349	0.7495	0.9173
		10	4645	110	409	300	0.9393	0.7881	0.9250
	C5.0	4	4404	86	433	541	0.8906	0.8343	0.8852
		10	4382	64	455	563	0.8861	0.8767	0.8852
Informed search: Simulated Annealing	RBF SVM	4	4656	121	398	289	0.9416	0.7669	0.9250
		10	4532	92	427	413	0.9165	0.8227	0.9076
	Random Forests	4	4414	95	424	531	0.8926	0.8170	0.8854
		10	4496	72	447	449	0.9092	0.8613	0.9046
	GBM	4	4524	110	409	421	0.9128	0.7881	0.9028
		10	4441	83	436	504	0.8981	0.8401	0.8926
	Linear SVM	4	4553	124	395	392	0.9207	0.7611	0.9056
		10	4384	85	434	561	0.8866	0.8362	0.8818
	*k*NN (k=7)	4	4443	114	405	502	0.8985	0.7803	0.8873
		10	4471	98	421	747	0.9041	0.8112	0.8953
	Naïve Bayes	4	4546	142	377	399	0.9193	0.7264	0.9010
		10	4595	135	384	350	0.9292	0.7399	0.9112
	C5.0	4	4413	101	418	535	0.8924	0.8054	0.8842
		10	4134	71	448	811	0.8360	0.8632	0.8386

**Table 5:** Summary of results using an ensemble of both proposals.

Ensemble Method	TP	FP	TN	FN	Sensitivity	Specificity	Accuracy
Top layer RBF SVM	4766	30	489	179	0.9638	0.9422	0.9617
Top layer C5.0	4746	24	495	199	0.9598	0.9538	0.9592
Logistic Regression WA	4708	19	500	237	0.9521	0.9634	0.9531
Top layer Naïve Bayes	4539	8	511	406	0.9179	0.9846	0.9242
Top layer Linear SVM	4441	9	510	504	0.8981	0.9827	0.9061
Top layer GBM	4426	9	510	519	0.8950	0.9827	0.9034
Top layer Random Forests	4419	7	512	526	0.8936	0.9865	0.9025
Top layer *k*NN (k=9)	4418	9	510	527	0.8934	0.9827	0.9019
